# *In Situ* Imaging of Candida albicans Hyphal Growth via Atomic Force Microscopy

**DOI:** 10.1128/mSphere.00946-20

**Published:** 2020-11-04

**Authors:** Arzu Çolak, Mélanie A. C. Ikeh, Clarissa J. Nobile, Mehmet Z. Baykara

**Affiliations:** a Department of Mechanical Engineering, University of California Merced, Merced, California, USA; b Department of Molecular and Cell Biology, University of California Merced, Merced, California, USA; University of Texas Health Science Center

**Keywords:** *Candida albicans*, atomic force microscopy, hyphal development

## Abstract

Candida albicans is an opportunistic fungal pathogen of humans known for its ability to cause a wide range of infections. One major virulence factor of C. albicans is its ability to form hyphae that can invade host tissues and cause disseminated infections. Here, we introduce a method based on atomic force microscopy to investigate C. albicans hyphae *in situ* on silicone elastomer substrates, focusing on the effects of temperature and antifungal drugs. Hyphal growth rates differ significantly for measurements performed at different physiologically relevant temperatures. Furthermore, it is found that fluconazole is more effective than caspofungin in suppressing hyphal growth. We also investigate the effects of antifungal drugs on the mechanical properties of hyphal cells. An increase in Young’s modulus and a decrease in adhesion force are observed in hyphal cells subjected to caspofungin treatment. Young’s moduli are not significantly affected following treatment with fluconazole; the adhesion force, however, increases. Overall, our results provide a direct means of observing the effects of environmental factors and antifungal drugs on C. albicans hyphal growth and mechanics with high spatial resolution.

**IMPORTANCE** Candida albicans is one of the most common pathogens of humans. One important virulence factor of C. albicans is its ability to form elongated hyphae that can invade host tissues and cause disseminated infections. Here, we show the effect of different physiologically relevant temperatures and common antifungal drugs on the growth and mechanical properties of C. albicans hyphae using atomic force microscopy. We demonstrate that minor temperature fluctuations within the normal range can have profound effects on hyphal cell growth and that different antifungal drugs impact hyphal cell stiffness and adhesion in different ways.

## Abstract (precis)

Candida albicans is one of the most common pathogens of humans. One important virulence factor of C. albicans is its ability to form elongated hyphae that can invade host tissues and cause disseminated infections. Here, we show the effect of different physiologically relevant temperatures and common antifungal drugs on the growth and mechanical properties of C. albicans hyphae using atomic force microscopy. We demonstrate that minor temperature fluctuations within the normal range can have profound effects on hyphal cell growth and that different antifungal drugs impact hyphal cell stiffness and adhesion in different ways.

## INTRODUCTION

Candida albicans is a commensal of humans that asymptomatically colonizes the skin, oral cavity, and gastrointestinal and reproductive tracts of healthy individuals (1, 2). C. albicans is also one of the most common opportunistic fungal pathogens of humans that typically causes superficial mucosal infections (e.g., vulvovaginal candidiasis) in healthy individuals (3). In immunocompromised individuals (e.g., AIDS patients, cancer and chemotherapy patients, and organ and bone marrow transplantation patients), C. albicans can also cause life-threatening bloodstream infections (1, 4). An important virulence trait of C. albicans is its ability to form biofilms on mucosal surfaces and on implanted medical devices, such as vascular and urinary catheters, cardiac valves, artificial vascular bypass devices, pacemakers, ventricular assist devices, dentures, and joint prostheses (5, 6). Biofilms are communities of microbial cells attached to a substratum or formed at a liquid-air interface that are embedded in a matrix of extracellular polymeric substances (6, 7). Biofilms can be highly tolerant and resistant to antifungal agents and host defenses (8, 9). C. albicans biofilm formation is a multifaceted process that begins when unicellular round yeast-form cells adhere to a surface (9, 10). This is followed by formation of elongated hyphal and pseudohyphal cells along with the production of the extracellular matrix (9–11). Hyphal cells are essential for biofilm formation and maintenance because they provide architectural support to the biofilm structure (6). They are also critical for C. albicans cells to invade epithelial cell layers and cause tissue damage in the host (12–16). Therefore, understanding how round yeast-form cells become elongated hyphal cells and how these cells grow and respond to external physical and chemical stimuli is fundamental to understanding how C. albicans can transform from a benign commensal to an invasive pathogen. By quantifying the variations in the physical and mechanical properties of hyphae with high spatial resolution as they develop, we can gain a comprehensive understanding of how C. albicans cells colonize and invade surfaces, such as host tissues and implanted medical devices. Moreover, considering the limited classes of antifungal drugs available in the clinic and the enhanced resistance of fungal biofilms to these antifungal drugs, the ability to directly observe the effects of antifungal drugs on hyphal growth with high spatial resolution could be instrumental in the development of new antifungal therapies.

Despite their importance, directly visualizing morphological transitions of microbial cells with high spatial resolution and quantifying mechanical properties of different cellular morphologies under physiological conditions are an outstanding challenge in microbiology. Current methods (i.e., those based on electron microscopy and optical microscopy) often (i) involve complex sample preparation procedures (such as drying and staining), (ii) require specialized environments (such as a vacuum) that can lead to the degradation of samples from their natural states, and (iii) do not provide structural and mechanical information simultaneously. On the other hand, unlike other microscopy techniques, atomic force microscopy (AFM) is a unique imaging method that not only enables surface topography imaging of living cells with detailed spatial resolution under relevant environmental conditions (e.g., in liquid media) but also probes mechanical properties (e.g., stiffness and adhesion) with piconewton resolution in force without the need for preparation steps such as dehydration and labeling, thus preserving the natural state of biological samples.

AFM has been used in prior studies for both high-resolution imaging and mechanical property characterization of various microorganisms (17–24), including the fungal species C. albicans (25–30). In these studies, AFM was used to investigate the native states of the living microbial cells of interest in aqueous solutions using artificial cell immobilization procedures, such as trapping in porous membranes (23, 31), hydrogels (32, 33), or microfabricated microwells (34, 35), to avoid the detachment or movement of cells. The entrapment of cells with such artificial methods, however, can induce mechanical stresses that could lead to cell damage and modification of growth rates and mechanical properties, thus potentially biasing measurements (36). For instance, the formation of C. albicans hyphal cells could not be observed in previous AFM studies using firmly attached yeast cells by mechanical entrapment, suggesting that cell function and/or viability may have been compromised in these studies due to the use of artificial trapping methods.

Despite the sizeable number of prior AFM studies that use C. albicans cells, there are only a few studies that specifically focus on hyphal cells (25, 28, 37–42). In these studies, hyphae were grown from a culture for several hours and then immobilized on a substrate using either chemical fixation or hydrophobic coatings, and measurements were performed at room temperature either in buffered medium or under dry conditions. The use of AFM, however, for the direct imaging of the dynamic process of C. albicans hyphal formation using cells that have naturally adhered to a clinically relevant substrate without the need for any artificial immobilization techniques is yet to be shown.

To address this critical gap in knowledge, here we introduce an AFM-based approach to investigate dynamic hyphal growth in C. albicans *in situ* using liquid medium on silicone elastomer substrates, a clinically relevant material used in intravascular catheters. First, we investigate the influence of temperature on hyphal growth by taking successive AFM measurements at several physiologically relevant temperatures. Furthermore, we use AFM to explore the effects of antifungal drugs (caspofungin and fluconazole) on hyphal cell growth as well as to assess the mechanical properties of hyphal cell walls (using Young’s [elastic] modulus and adhesion force) following treatment with antifungals. All of our experiments are performed at physiologically relevant temperatures of the human body and thus address a crucial drawback of prior C. albicans AFM studies that were performed at room temperature. Our results constitute the first direct *in situ* AFM study of unrestricted hyphal growth in C. albicans performed under physiologically relevant conditions and in the presence of commonly prescribed antifungal drugs, thus setting the framework for future mechanistic studies.

## RESULTS

### Effects of physiologically relevant temperatures on hyphal cell growth determined by AFM.

**FIG 1:**
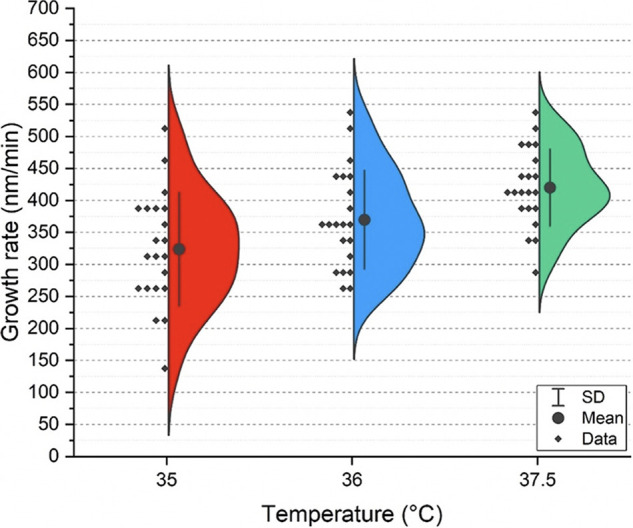
The effect of temperature on hyphal growth rates. Semiviolin plots depicting C. albicans hyphal growth rates with changing physiologically relevant temperatures in Spider medium.

We investigated the effect of temperature on growing hyphal cells (starting at germination) using successive AFM scans. The cells were scanned while growing on silicone elastomer substrates in Spider medium, a hyphal cell-inducing medium, at the physiologically relevant temperatures of 35°C, 36°C, and 37.5°C. For each temperature, 21 different hyphal cells, each from different culture batches, were scanned by AFM in tapping mode, and their growth rates were calculated (see Materials and Methods). Figure 1 shows the hyphal growth rate distributions in Spider medium at the three different temperatures assessed. The mean growth rate values were measured as 323.8 nm/min, 369.9 nm/min, and 420.1 nm/min at 35°C, 36°C, and 37.5°C, respectively.

### Effects of antifungal drugs on hyphal cell growth determined by AFM.

**FIG 2:**
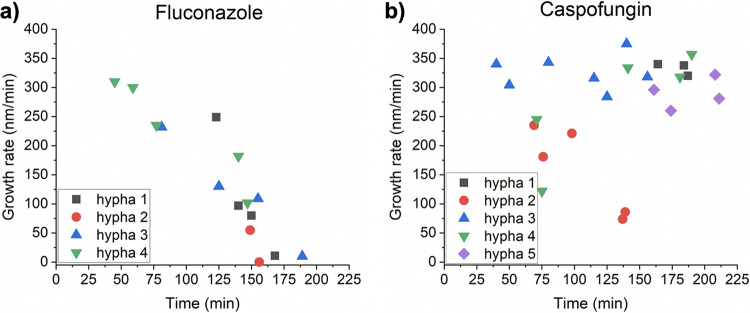
The effect of antifungal drugs on hyphal growth rates. (a) The evolution of growth rates in the presence of fluconazole. (b) The evolution of growth rates in the presence of caspofungin.

Since antifungal drugs are routinely used to treat C. albicans infections, we used AFM to investigate the growth of hyphal cells treated with fluconazole and caspofungin, two of the most commonly used antifungal drugs in the clinic. For these studies, newly germinated hyphal cells were grown on silicone elastomer substrates on the AFM sample stage for 20 min in Spider medium without drugs at 37.5°C. Spider medium without antifungal drugs was then exchanged for Spider medium containing 0.5 ng/ml of the antifungal drug fluconazole or caspofungin. Growth of hyphal cells (*n* = 4 cells for cells grown in the presence of fluconazole and *n* = 5 cells for cells grown in the presence of caspofungin, all from different starting cultures) was observed via consecutive AFM scans in tapping mode for 3 h at 37.5°C. Our results using hyphal cells demonstrate that they are more susceptible to fluconazole treatment (Fig. 2a) than caspofungin treatment (Fig. 2b). The growth rate of hyphal cells treated with fluconazole gradually decreased over time and nearly stopped after 3 h in the presence of fluconazole. When cells were exposed to caspofungin, however, few changes in overall hyphal growth rates were observed. Only one out of five hyphal cells showed a decrease in growth. Furthermore, compared to the ∼420-nm/min average growth rate of hyphal cells at 37.5°C in drug-free medium (see the green semiviolin distribution in Fig. 1), in the presence of either antifungal drug (fluconazole or caspofungin), the average growth rate of hyphal cells was lower (∼300 to 350 nm/min) after just ∼40 min of drug exposure (Fig. 2a and b, ∼40-min time point).

### Hyphal cell wall stiffness in the presence of antifungal drugs.

**FIG 3:**
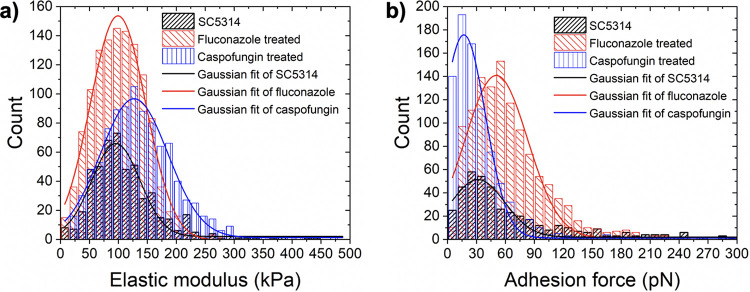
The effect of antifungal drugs on the mechanical properties of hyphal cell walls. Distribution of Young’s (elastic) moduli (a) and adhesion forces (b) recorded on C. albicans hyphal cells prior to antifungal drug exposure (black bars) and after exposure to fluconazole (red bars) or caspofungin (blue bars).

We next hypothesized that treatment of C. albicans with caspofungin or fluconazole would alter hyphal cell wall mechanics. Quantitative measurements of caspofungin- or fluconazole-induced mechanical variations on C. albicans hyphal cells have not been previously reported through the use of AFM. To test our hypothesis, we measured Young’s moduli of growing C. albicans hyphal cells using AFM force-distance spectroscopy after treatment with caspofungin or fluconazole for 2 h at a concentration of 0.5 ng/ml. For each antifungal treatment, Young’s moduli were determined as shown in Fig. 3a by fitting a Gaussian function to the overall histogram composed of all modulus values calculated from maps of 20 × 20 force-distance curves recorded on three different C. albicans hyphal cells grown from two independent cultures. We found that the mean Young’s modulus of C. albicans hyphal cells had a value of 95.7 kPa (total number of recorded force curves, *n* = 505) prior to antifungal treatment, which is similar to previously published results (26, 43). After caspofungin treatment, the mean Young’s modulus of C. albicans hyphal cells was increased by ∼30% to a value of 127 kPa (*n* = 954). On the other hand, fluconazole-treated hyphal cells had nearly identical stiffness as untreated hyphal cells with a mean Young’s modulus of 99.4 kPa (*n* = 1,195).

### Adhesion force measurements of hyphal cells in the presence of antifungal drugs.

To determine whether treatment with caspofungin or fluconazole can affect the strength of hyphal cell adherence, adhesion force measurements were performed on hyphal cells using AFM by recording maps of 20 × 20 force-distance curves. Three different C. albicans hyphal cells from two independent cultures were measured at 37.5°C after 2 h of treatment with caspofungin or fluconazole at concentrations of 0.5 ng/ml. Figure 3b shows the distribution of the measured adhesion forces of the C. albicans hyphal cells before and after treatment with the two antifungal drugs. The mean adhesion force of hyphal cells without antifungal treatment (total number of recorded force curves, *n* = 504) was 31.5 pN. After treatment with fluconazole, the mean adhesion force increased to 50.6 pN (*n* = 1195). In contrast, a decrease in the mean adhesion force to a value of 16.9 pN (*n* = 884) was observed for hyphal cells treated with caspofungin. Changes in adhesion induced by both antifungal drugs were statistically significant (*P* < 0.0001; calculated via Tukey’s *post hoc* comparisons). We note that a direct comparison of adhesion forces reported here with those in the literature is not feasible given that bare AFM probes were utilized in our study whereas published results were obtained with probes functionalized with certain cells (38–40) or molecules (26, 27, 44).

### Surface topography measurements of hyphal cells in the presence of antifungal drugs.

To explore the effects of antifungal treatment on the surface topography of hyphal cells, we performed high-resolution AFM scans on hyphal cells in the presence and absence of caspofungin and fluconazole as reported in Fig. S1 in the supplemental material. We observed no significant changes in surface roughness or topography of hyphal cell walls upon treatment with fluconazole or caspofungin at the antifungal concentration used (0.5 ng/ml) (Fig. S1).

10.1128/mSphere.00946-20.1 FIG S1 AFM topography images of C. albicans hyphal cell walls recorded in Spider medium at 37°C, prior to drug exposure (a to c), after 0.5-ng/ml caspofungin exposure for 2 h (d to f), and after 0.5-ng/ml fluconazole exposure for 2 h (g to i) (color scale ranges: 1.60 μm, 138.9 nm, 26 nm, 1.75 μm, 248 nm, 21.5 nm, 1.60 μm, 235.5 nm, and 38.5 nm, respectively). The calculated root-mean-square (RMS) roughness value from high-resolution AFM topography images (c, f, and i) was 2.9 nm for cells without drug exposure and 2.2 nm and 2.7 nm for cells exposed to caspofungin and fluconazole, respectively. The red boxes highlight the locations of areas scanned in the subsequent image. Download FIG S1, TIF file, 0.8 MB. Copyright © 2020 Çolak et al. 2020 Çolak et al. This content is distributed under the terms of the Creative Commons Attribution 4.0 International license.

## DISCUSSION

The ability of C. albicans cells to transition from yeast to hyphal forms is influenced by many environmental cues, including serum, *N*-acetylglucosamine, pH, carbon source, oxygen levels, and temperature (45–49). Hyphal cells are a critical component of C. albicans biofilms and are the morphological form that invades host epithelial cell layers to cause tissue damage and infections (50). Given the importance of the yeast-to-hyphal transition for virulence in the host, we investigated the effects of temperature on growing hyphal cells. Our results indicate that hyphal growth rates in Spider medium increase significantly with each incremental increase in temperature. Although this finding is not surprising given that physiological temperatures are known to induce hyphal formation, this is the first study to quantitatively evaluate such profound effects on hyphal growth as incremental physiological temperature adjustments occur. Given that the normal human body temperature fluctuates within these temperature ranges over the course of a 24-h period (51, 52), our findings indicate that minor body temperature changes within the normal range can markedly affect the morphology of C. albicans and thus significantly affect its pathogenic potential.

We also used AFM to investigate the growth of hyphal cells treated with fluconazole and caspofungin and found that hyphal cells are more susceptible to fluconazole than to caspofungin. The observation that fluconazole is more effective at suppressing hyphal growth compared to caspofungin is perhaps not surprising given that azoles block the synthesis of ergosterol, a major component of the fungal cell membrane (53). Caspofungin, on the other hand, inhibits the synthesis of cell wall β-1,3-d-glucans (54, 55) and may play a more central role in maintaining mechanical rigidity of the fungal cell wall. Therefore, we hypothesized that treatment of C. albicans with caspofungin would alter hyphal cell wall mechanics. There is some controversy in the literature regarding the mechanisms by which caspofungin impacts the mechanical properties of C. albicans. In one AFM study performed on C. albicans yeast-form cells, treatment with caspofungin caused a softening of the cell wall (44), while other studies (26, 43) showed that Young’s modulus, a measurement of stiffness, was increased following the treatment of C. albicans yeast-form cells with caspofungin. Based on the results we report here, we found that the mean Young’s modulus of C. albicans hyphal cells was increased by ∼30% after caspofungin treatment whereas fluconazole-treated hyphal cells had nearly identical stiffness as untreated hyphal cells. In C. albicans, inhibition of β-1,3-d-glucans by caspofungin treatment is known to result in an increase in the synthesis of chitin (56), a major polysaccharide that contributes to cell wall strength and can compensate for mechanical perturbations. Therefore, the cell wall stiffening we observe after caspofungin treatment is likely attributed to an increase in the amount of chitin in the hyphal cell wall (26, 43).

Other than the ability of C. albicans cells to undergo the yeast-to-hyphal transition, the ability of C. albicans cells to adhere to surfaces and form biofilms is another important virulence trait of this common human fungal pathogen. Adherence to surfaces is the first step in biofilm formation and is required for C. albicans cells to colonize host tissues and implanted medical devices (6, 28). As such, we investigated the effect of caspofungin and fluconazole treatments on adhesion measured via AFM on C. albicans hyphal cells. Our finding suggests that by significantly decreasing the adhesion forces of C. albicans cells attached to a surface, caspofungin is likely to be more effective than fluconazole in treating C. albicans biofilm infections in the clinic. Indeed, it is known that of the three major antifungal drugs commonly used in the clinic (caspofungin, fluconazole, and amphotericin B), caspofungin is the most effective against preformed C. albicans biofilms grown *in vitro*, while fluconazole is completely ineffective (57, 58).

It is feasible that differences in adhesion strength could be caused by drug-induced changes to the topography of the hyphal cell surface. The idea that caspofungin can cause changes to the C. albicans cell surface has been established in prior studies (44, 59, 60). However, we observed no significant changes in surface roughness or topography of hyphal cell walls upon treatment with fluconazole or caspofungin at the antifungal concentration used (see Fig. S1 in the supplemental material). It is possible that these antifungal drug concentrations were not adequate to induce damage to the hyphal cell walls and change the adhesion forces in that fashion.

In the absence of topographical changes that would explain the measured differences in C. albicans hyphal cell adhesion, the adherence of C. albicans cells to surfaces is likely mediated predominantly by adhesion molecules (adhesins) on the cell surface, such as the Als (agglutinin-like sequence) glycosylphosphatidylinositol (GPI)-anchored proteins (15, 61, 62). In fact, it was recently shown using AFM single-molecule analyses that high Als3 levels on the surface of hyphal cells promote cell adherence (37, 40). Taken together with our findings, it seems plausible that changes in adhesion forces upon treatment with caspofungin and fluconazole could correspond to variations in the expression of adhesin levels on the surfaces of hyphal cells. Nevertheless, further work needs to be performed at the single-molecule level to fully understand the physical and molecular reasons behind the effects of antifungal drugs on adhesion strength.

Finally, we note that the antifungal drug concentrations employed in this study are below the clinically relevant MIC values for caspofungin and fluconazole. These concentrations were chosen because they represent the highest antifungal drug concentrations that did not induce detachment of the fungal cells from the substrate. Despite this fact, our results demonstrate that even concentrations well below the MICs for these antifungal drugs have a measurable impact on hyphal growth and the mechanical properties of hyphal cells. This new information was not uncovered in previous AFM studies that solely focused on mechanically trapped yeast cells.

Here, we introduced for the first time the application of AFM for the *in situ* characterization of the growth dynamics and mechanical properties of C. albicans hyphal cells grown without entrapment on a clinically relevant material (silicone) at physiologically relevant temperatures. We showed that incremental changes in normal human body temperatures from 35°C to 37.5°C can have significant effects on hyphal growth rates. Given that the normal human body temperature fluctuates within these temperature ranges over the course of a 24-h period, our findings indicate that minor body temperature changes within the normal range can markedly affect the morphology of C. albicans and thus significantly affect its pathogenic potential.

We also explored by AFM the effects of the most widely used antifungal drugs to treat C. albicans infections, caspofungin and fluconazole, on hyphal growth under physiologically relevant conditions. Our results showed that although caspofungin and fluconazole exposure did not significantly alter hyphal cell surface topography at concentrations of 0.5 ng/ml, treatment with fluconazole at the same concentration caused hyphal cell growth to cease after 3 h, while caspofungin had little to no impact on hyphal growth rates. Exposure to caspofungin and fluconazole also had different effects on the mechanical properties of hyphal cells. Hyphal cells exposed to caspofungin had increased stiffness and decreased adhesive strength relative to untreated hyphal cells and to fluconazole-treated hyphal cells. Interestingly, exposure to fluconazole also causes C. albicans hyphal cells to have increased adhesive strength relative to untreated hyphal cells. Overall, our results indicate that fluconazole and caspofungin have different effects on the growth and mechanical properties of C. albicans hyphal cells, which could impact the pathogenic potential of C. albicans in a host. These results also suggest that the combined or sequential use of fluconazole and caspofungin during treatment of C. albicans infections in the clinic could be beneficial when either drug used independently is ineffective. Overall, our findings set the stage for future studies aimed at uncovering the underlying physical and molecular mechanisms of the effects of temperature and antifungal drugs on C. albicans cells that could be further pursued with methods such as single-molecule force spectroscopy using AFM.

## MATERIALS AND METHODS

### Growth of C. albicans cells.

C. albicans clinical reference strain SC5314 was used for all experiments. SC5314 was grown for 2 days at 30°C on yeast extract-peptone-dextrose (YPD) agar (1% yeast extract, 2% Bacto peptone, 2% dextrose, 2% agar). To prepare liquid cultures, a single colony was inoculated into YPD liquid medium and incubated overnight at 30°C with agitation at 225 rpm. C. albicans cells were harvested by centrifugation, washed three times with sterile phosphate-buffered saline (PBS) (pH 7.2), and resuspended in 1 ml of PBS buffer. Optical densities at 600 nm (OD_600_) were measured and adjusted to ∼19, and a 1:2,000 dilution of the cells was made in PBS. This dilution factor was chosen because it maintained a uniform distribution of cells on the silicone substrate at a low-enough density for proper AFM imaging.

### Preparation of C. albicans cells on silicone elastomer substrates for AFM.

To prevent cell detachment during AFM scans, squares of silicone elastomer substrates (1.5 by 1.5 cm) were cut from silicone sheets (Cardiovascular Instrument Corp.; PR72034-060N) and precoated with bovine serum as follows: silicone squares were washed in distilled and deionized water, dried, autoclaved, placed in sterile 12-well polystyrene non-tissue culture-treated plates (Corning; 351143), and then incubated with 2 ml of 1× fetal bovine serum (FBS) (Sigma-Aldrich) for 60 min at 37°C.

An 0.5-ml amount of the C. albicans cell solution was inoculated in 2 ml sterile Spider medium (63) (1% nutrient broth, 1% mannitol, 1% K_2_PO_4_, pH 7.2) at 37°C. The bovine serum-coated silicone elastomers were added into the C. albicans Spider medium cell solution and incubated for 20 min at 37°C with agitation at 200 rpm in an ELMI digital thermostatic shaker. At the end of this procedure, C. albicans cells initiated hyphal formation (germ tubes were on the order of 5 μm in length) on the silicone elastomer substrates (see Fig. S2).

10.1128/mSphere.00946-20.2 FIG S2 (a and c) AFM height images of C. albicans yeast-form cells with induced germ tubes cultivated on silicone elastomer substrates in Spider medium at 37°C (color scale ranges: 2.8 μm and 3.0 μm, respectively). (b and d) Corresponding phase images (color scale ranges: 44° and 55°, respectively). Download FIG S2, TIF file, 0.7 MB. Copyright © 2020 Çolak et al. 2020 Çolak et al. This content is distributed under the terms of the Creative Commons Attribution 4.0 International license.

### Imaging the growth of C. albicans hyphal cells by AFM.

Surface topography scans and growth rate measurements of C. albicans hyphal cells were carried out with an Asylum Research Cypher VRS atomic force microscope (Santa Barbara, CA, USA). Liquid Spider medium at temperatures varying between 35°C and 37.5°C was used, depending on the specific experiment. Silicon nitride cantilevers with silicon tips (Olympus; BL-AC40TS; nominal spring constant *k* = 0.1 N/m) were used in all experiments. Prior to the experiments, the normal spring constant of each cantilever was determined from the first resonance frequency (which was around 27 kHz in buffer solution) of the thermal noise spectra using the GetReal calibration procedure integrated into the AFM software.

The silicone elastomer substrate with C. albicans germ tubes was attached to a steel sample puck using double-sided tape. The mounted elastomer was transferred onto the heating stage of the AFM, which was already set to a temperature between 35°C and 37.5°C, depending on the experiment. The silicone substrate containing C. albicans germ tubes was washed 3 times with 100 ml Spider medium to remove unattached cells. Twenty milliliters of Spider medium at 37°C was drop-cast onto the silicone surface before performing topographical scans via tapping mode of AFM in liquid. Once a suitable region with cells that had initiated hyphal formation was found with the optical camera of the AFM, the cantilever was approached, and an initial scan was made over an area of 25 μm by 25 μm with a scan rate of 0.30 Hz. A consecutive scan of the same area was then performed at the same scan speed. Figure S3 shows representative, consecutive topographical images of a growing C. albicans hyphal cell recorded as described above.

10.1128/mSphere.00946-20.3 FIG S3 (a and b) AFM topography images of a C. albicans hyphal cell grown on a bovine serum-coated silicone elastomer substrate, recorded via tapping mode in Spider medium in the form of two consecutive scans separated by 34 minutes and 12 seconds (color scale range: 3.7 μm). The yellow arrows show the scan directions. (c) Comparison of the height profiles of the hyphal cell along the solid white lines indicated in panels a and b. Download FIG S3, TIF file, 0.7 MB. Copyright © 2020 Çolak et al. 2020 Çolak et al. This content is distributed under the terms of the Creative Commons Attribution 4.0 International license.

### Calculating the growth rates of C. albicans hyphal cells.

After AFM images were processed with the scanning probe microscopy data analysis software package Gwyddion 2.53 (64), height profiles along the longitudinal directions of a particular hyphal cell were studied in successive AFM scans (see Fig. S3) to calculate hyphal growth in terms of the apex-to-apex distance between the line profiles being compared, as shown representatively in Fig. S3c. The elapsed time associated with apex-to-apex growth of a hyphal cell was calculated from the total number of scanned lines between apexes in consecutive AFM scans and the scan time per line. Finally, the growth rate was calculated as the ratio of the growth between apexes in AFM scans and the time elapsed to scan the apex-to-apex growth by AFM.

For the hyphal cell shown as an example in Fig. S3, the scan rate was 0.25 Hz (i.e., each image was composed of 256 lines recorded in 17.1 min). Therefore, apex-to-apex growth of the hyphal cell in consecutive scans was recorded in 27.7 min for 414 scanned lines (i.e., 202 lines from Fig. S3a and 212 lines from Fig. S3b). Hence, the growth rate of this hyphal cell is 300 nm/min for a total of 8,300 nm of growth, calculated from the comparison of line profiles in Fig. S3c.

### Measuring adhesion forces and Young’s moduli of C. albicans hyphal cells via AFM force-distance spectroscopy.

Prior to force measurements, substrates were briefly imaged in tapping mode, first over a large area (e.g., 30 μm in lateral size) to find hyphal cells, and then over a small area (e.g., 400 nm in lateral size) to focus on a location at the middle regions of a growing hyphal cell (see Fig. S4). To probe adhesion forces and elastic moduli of hyphal cells, maps of 20 × 20 force-distance curves were recorded on scanned areas (see Fig. S5) of the hyphal cell with an Asylum Research Cypher VRS atomic force microscope (Santa Barbara, CA, USA) in Spider medium at 37°C with BL-AC40TS cantilevers (Olympus; *k* = 0.1 N/m, *f* = 27 kHz in liquid), which were calibrated prior to the experiments as described above. To record the interaction forces between the AFM probe and the hyphal cell of interest as a function of the distance between them, the probe was approached to and then retracted from the hyphal cell at a constant speed of 1,000 nm/s. Force-distance curves were recorded on three different hyphal cells from two independent cultures of C. albicans, with a maximum applied force of 250 pN to ensure the indentation was lower than 10% of hyphal cell height to avoid cross talk with the mechanical properties of the substrate (65).

10.1128/mSphere.00946-20.4 FIG S4 Schematic demonstration of a representative measurement on a C. albicans hyphal cell used for elastic modulus and adhesion force measurements: (a) side view and (b) top view. Download FIG S4, TIF file, 0.2 MB. Copyright © 2020 Çolak et al. 2020 Çolak et al. This content is distributed under the terms of the Creative Commons Attribution 4.0 International license.

10.1128/mSphere.00946-20.5 FIG S5 Representative maps of 20 × 20 force-distance curves used for calculating Young’s moduli (a to c) and adhesion forces (d to f), recorded at 37°C in Spider medium for C. albicans hyphal cells growing without antifungal drugs (a and d) and after 0.5-ng/ml fluconazole (b and e) or caspofungin (c and f) exposure. All images are the same size. Corresponding histograms are shown in the main text for Young’s moduli (Fig. 3a) and for adhesion forces (Fig. 3b). Download FIG S5, TIF file, 0.4 MB. Copyright © 2020 Çolak et al. 2020 Çolak et al. This content is distributed under the terms of the Creative Commons Attribution 4.0 International license.

To convert AFM force-distance curves to force-indentation curves, each force-distance curve on the force maps was processed by correcting the baseline offset and tilt of the curves and by subtracting the effects of cantilever bending using the Igor Pro software (WaveMetrics Inc.). From the force-indentation curves, the adhesion force of each curve was calculated as the lowest negative rupture force recorded when retracting the tip from the surface of the hyphal cell of interest. The measured adhesion force values are presented as pixelated maps in Fig. S5d to f, and statistical distributions for these values are shown in Fig. 3b. The mean calculated adhesion force of each statistical distribution was determined by fitting a Gaussian function to the overall histogram composed of all measured adhesion force values.

The elastic stiffness of hyphal cells can be deduced from the approach segment of the force-indentation curves. After processing the force-distance curves, the characteristic power-law shape of the approach segment was fitted to the Hertz model (66) for a paraboloid tip geometry to estimate Young’s modulus, *E*. The Hertz model describes the deformation behavior of purely linear elastic materials (67). Biological samples are never perfectly linearly elastic but are instead viscoelastic with a short range of linear elasticity (68). Several studies have shown that for small indentation depths and for small indenters compared to sample dimensions, the force-indentation data follow Hertzian mechanics (69–71). Therefore, to ensure data are extracted from the linearly elastic regime, experiments were performed for shallow indentation depths up to 100 nm with a maximum force of 250 pN. The results are shown as pixelated two-dimensional (2D) maps in Fig. S5a to c, and statistical distributions of Young’s moduli values are shown in Fig. 3a.

### Data availability.

The data that support the findings of this study are available from the corresponding authors upon request.
