# Supplementary figures and images for: In Situ Imaging of Candida albicans Hyphal Growth via Atomic Force Microscopy
Source: mSphere. 2020 Nov 4;5(6):e00946-20. doi: 10.1128/mSphere.00946-20 (PMC7643834; doi:10.1128/mSphere.00946-20)

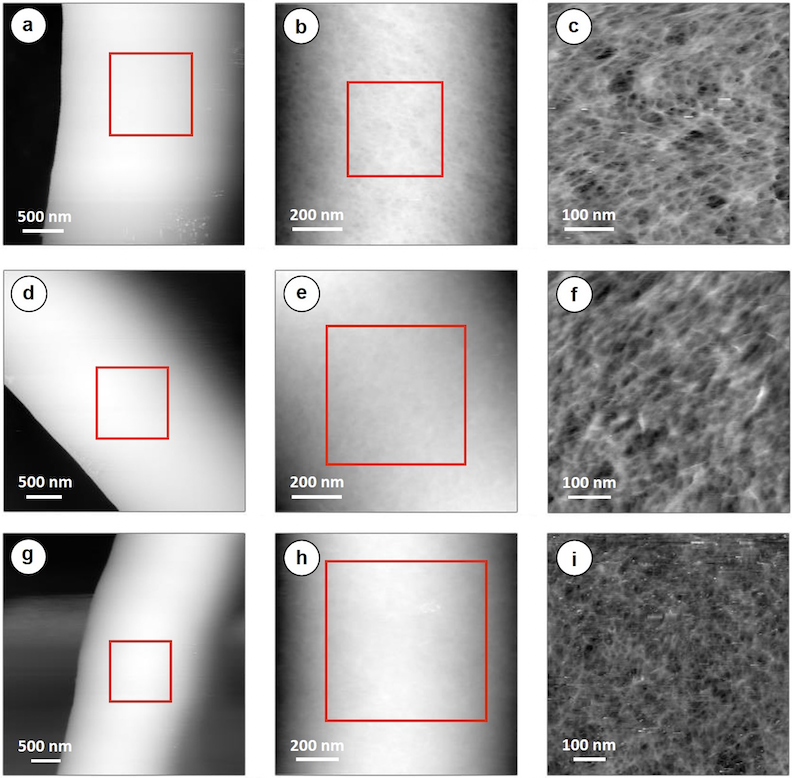

Supplement: FIG S1 [file mSphere.00946-20-sf001.tif]

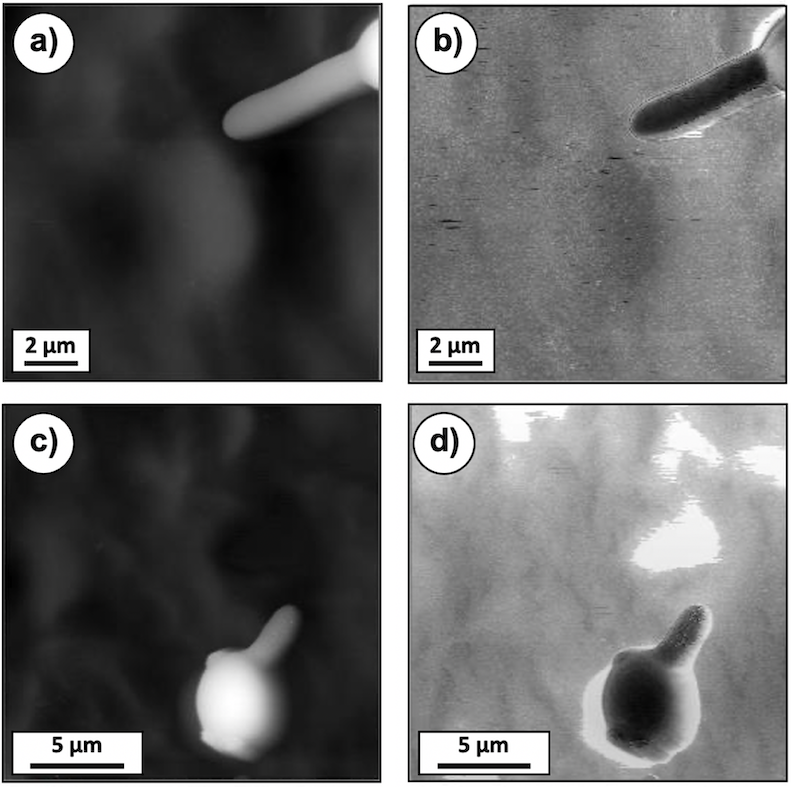

Supplement: FIG S2 [file mSphere.00946-20-sf002.tif]

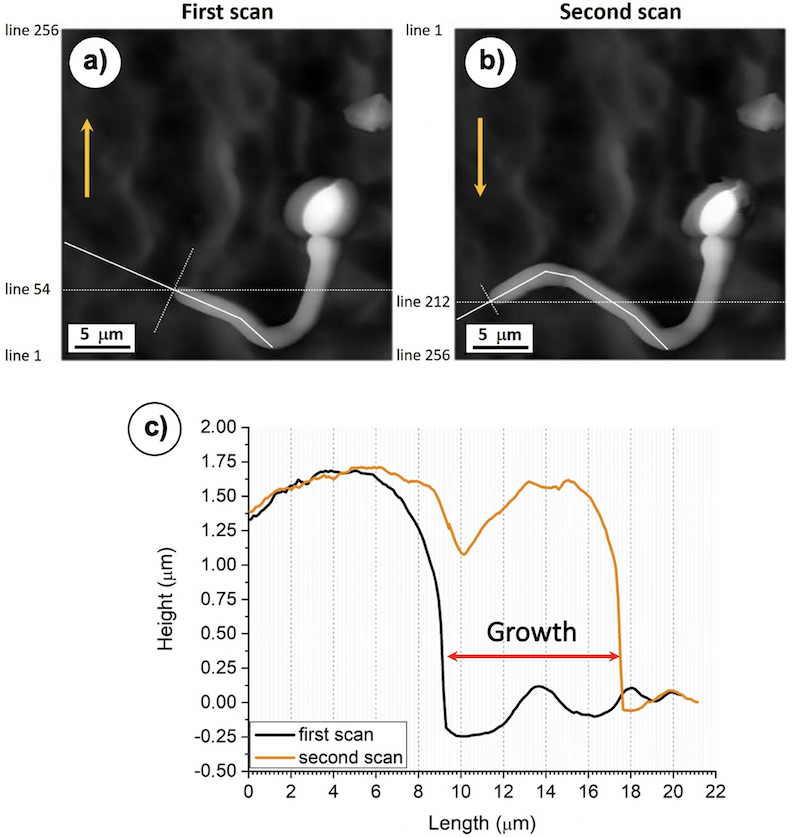

Supplement: FIG S3 [file mSphere.00946-20-sf003.tif]

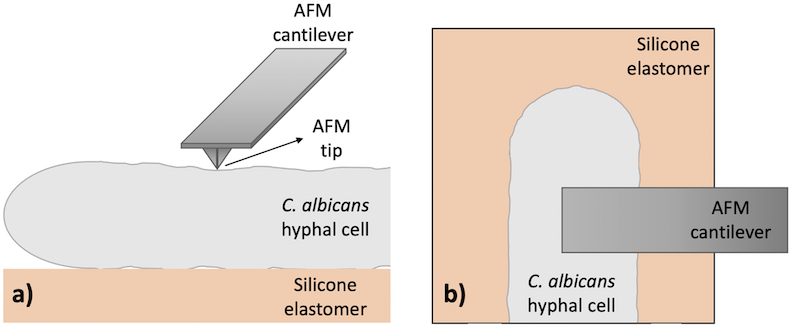

Supplement: FIG S4 [file mSphere.00946-20-sf004.tif]

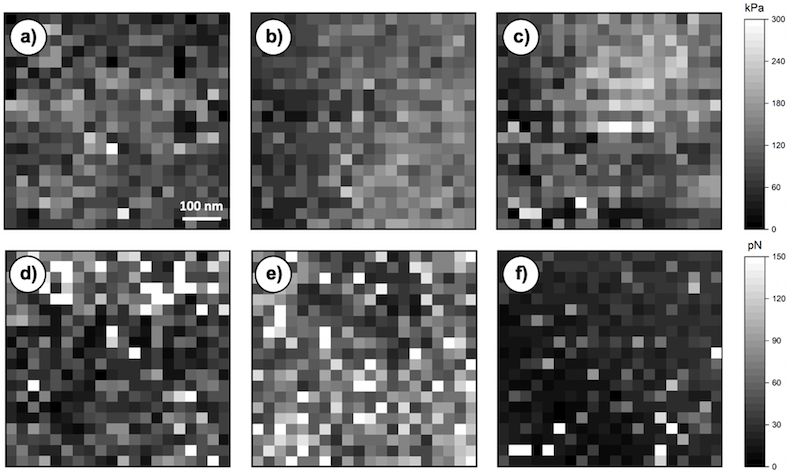

Supplement: FIG S5 [file mSphere.00946-20-sf005.tif]
